# Hip fracture surgery within 24 hours: no association between time to surgery and mortality in a real-world cohort

**DOI:** 10.1007/s00068-026-03331-0

**Published:** 2026-09-15

**Authors:** Carlos Pankratz, Malte Wolters, Raffael Cintean, Florian Gebhard, Konrad Schuetze, Alexander Eickhoff

**Affiliations:** Department of Trauma-, Hand-, Plastic- and Reconstructive Surgery University Ulm Medical Centre, Albert-Einstein-Allee 23, 89081 Ulm, Germany

**Keywords:** Hip fracture, Time to surgery, Mortality, Early surgical care, Outcome, Perioperative complications

## Abstract

**Purpose:**

Early surgery is recommended for hip fractures, and in Germany, operative treatment within 24 h has been mandatory since 2021. While delays beyond 24–48 h are associated with worse outcomes, it remains unclear whether further acceleration within a 24-hour timeframe improves survival. This study evaluated mortality and perioperative risk factors within a strictly implemented early-treatment pathway.

**Methods:**

This retrospective single-centre cohort study included 1,051 consecutive patients with femoral neck and trochanteric fractures treated surgically within 24 h of admission (2016–2020). Time to surgery was grouped into < 6 h, 6–12 h, and > 12–24 h. The primary outcome was one-year mortality; secondary outcome was two-year mortality. Mortality data were obtained from the national citizen registry. Multivariable logistic and Cox proportional hazards regression analyses were performed for one-year mortality.

**Results:**

Mean time to surgery was 10.2 ± 6.2 h. One-year mortality was 29.5% and increased to 40.1% at two years. Mortality did not differ significantly between time-to-surgery groups (< 6 h: 30.9%, 6–12 h: 26.4%, > 12–24 h: 31.5%; *p* = 0.258). This finding was consistent across all adjusted analyses. In contrast, non-surgical complications were the strongest independent predictors of mortality, particularly pneumonia (OR 2.1), renal failure (OR 5.2), and cardiac events (OR 5.1). Surgical complications and implant choice were not associated with mortality.

**Conclusion:**

Within a 24-hour treatment pathway, further reduction of time to surgery was not associated with improved survival in this cohort. Mortality was strongly associated with perioperative medical complications. These findings support current early-surgery targets but suggest that optimisation of perioperative and orthogeriatric care may be at least as important for improving outcomes as further reductions in time to surgery.

**Supplementary Information:**

The online version contains supplementary material available at https://doi.org/10.1007/s00068-026-03331-0.

## Introduction

Hip fractures, comprising femoral neck and trochanteric fractures, represent one of the most common and severe injuries in the elderly population and are associated with substantial morbidity, mortality, and healthcare costs worldwide [1]. With ageing populations, the incidence of these fractures continues to rise, posing an increasing challenge to healthcare systems across Europe and beyond [2]. Despite advances in surgical techniques and perioperative management, one-year mortality after hip fracture remains substantial, consistently exceeding 20% in contemporary cohorts [3]. In a previously published cohort of exclusively geriatric patients from our institution, one-year mortality exceeded 35%, highlighting the particular vulnerability of frail elderly patients [4].

Timely surgical intervention has long been regarded as a key modifiable factor influencing outcomes after hip fractures [5, 6]. Several international studies suggest that early surgery may be associated with reduced mortality and lower complication rates, particularly among patients with a high burden of comorbidities [7, 8]. However, most of the existing evidence has focused on comparisons between surgery performed within versus beyond 24–48 h after admission. Whether surgery performed very early within an established 24-hour treatment framework confers additional survival benefit remains unclear.

The optimal timing of surgery remains a matter of ongoing debate. Most contemporary guidelines recommend early operative management, typically within 24 to 48 h after hospital admission. For example, the British National Institute for Health and Care Excellence advises surgery “on the day of, or the day after, admission” [9], while the American Academy of Orthopaedic Surgeons states that “surgery within 24–48 hours of admission may be associated with better outcomes” [10]. In Germany, a paradigm shift occurred in 2021 with the introduction of mandatory national quality regulations requiring operative treatment of hip and proximal femur fractures within 24 hours of hospital admission [11]. These regulations aim to standardise care and improve outcomes but also impose significant logistical and clinical challenges, particularly in elderly patients with complex medical conditions or ongoing antithrombotic therapy. However, direct comparative data evaluating outcomes across different time-to-surgery intervals within a strictly implemented 24-hour treatment window are essentially lacking.

Therefore, the aim of the present study was to analyse mortality and complication rates in a large retrospective cohort of patients with hip fractures treated within 24 h after admission. Specifically, we aimed to investigate whether different time-to-surgery intervals within this 24-hour window were associated with differences in one-year mortality and to identify perioperative factors independently associated with adverse outcomes. By using registry-based mortality data with complete ascertainment of vital status for all included patients, this study provides a comprehensive real-world assessment of survival within a strictly applied 24-hour treatment model.

## Methods

This study was designed as a retrospective single-centre cohort study conducted at a level-one trauma centre. Our institution has an established internal treatment pathway aiming for surgical management of hip fractures within 24 h for several years. The study was approved by the local institutional ethics committee, and all procedures were conducted in accordance with the Declaration of Helsinki. Due to the retrospective design, the requirement for informed consent was waived.

**Fig. 1:**
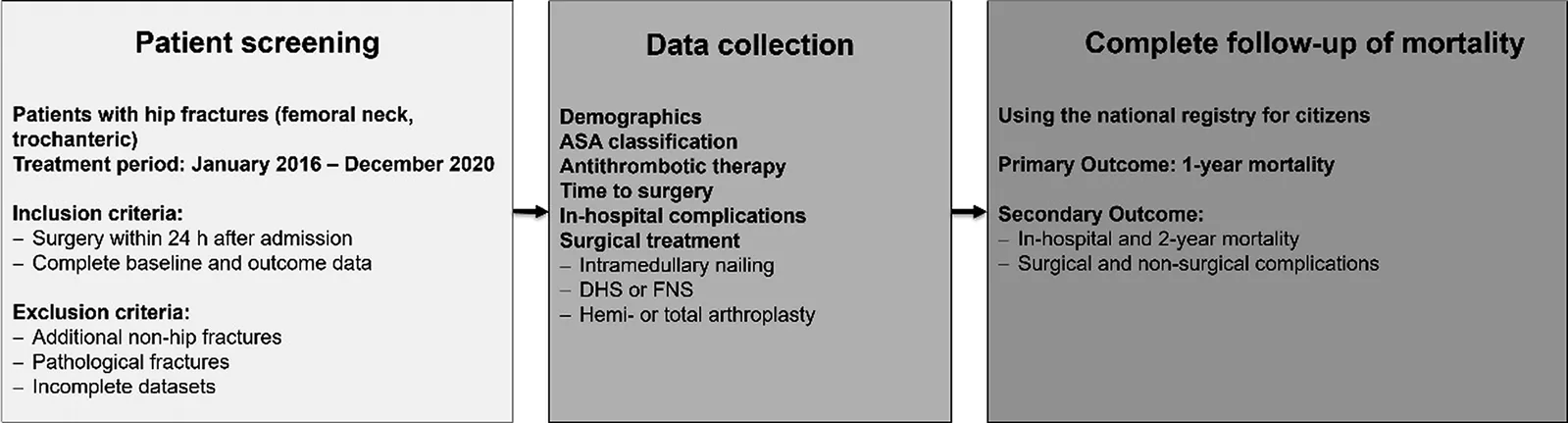
Study design retrospective single-centre cohort study. ASA: American Society of Anesthesiologists; DHS: Dynamic Hip Screw; FNS: Femoral Neck System

All consecutive patients treated between January 2016 and December 2020 for hip fractures, defined as femoral neck and trochanteric fractures, were screened for eligibility. Inclusion criteria were operative treatment within 24 h after hospital admission and availability of complete baseline and outcome data. Admission was defined as the time of first documented registration at our institution. For patients transferred from other hospitals, time to surgery was calculated from admission to our institution rather than from presentation at the referring hospital. Patients with fractures outside the defined anatomical regions, pathological fractures, periprosthetic fractures, or with incomplete datasets were excluded (Fig. 1).

Surgical treatment was performed according to fracture type and surgeon discretion and included intramedullary nailing, dynamic hip screw or femoral neck system (DHS/FNS) fixation, and hemi- or total hip arthroplasty. Clinical data were obtained through a structured retrospective review of electronic medical records. Collected variables included age, sex, fracture type, American Society of Anesthesiologists (ASA) classification, treatment modality, antithrombotic medication, time to surgery, and in-hospital complications. Time to surgery was defined as the interval between hospital admission and skin incision and was recorded in minutes. Surgical complications were defined as implant- or procedure-related adverse events requiring operative revision, including secondary displacement, dislocation, implant failure, postoperative hematoma, and surgical site infection. Non-surgical complications comprised urinary tract infection, pneumonia, thrombosis, renal failure, and cardiac events occurring during the in-hospital stay. Pneumonia was defined as an in-hospital diagnosis documented in the medical record based on clinical signs, radiological findings, and initiation of antibiotic treatment. Renal failure was defined as an acute deterioration of renal function documented in the medical record during the in-hospital stay and requiring medical management. Cardiac events were defined as a composite endpoint including myocardial infarction (ST-elevation and non-ST-elevation myocardial infarction), clinically relevant cardiac arrhythmias requiring treatment, and decompensated heart failure.

The primary outcome was one-year mortality. Secondary outcome was two-year mortality. Mortality at one and two years was retrieved from the national citizen registry, ensuring complete follow-up and complete ascertainment of vital status for all included patients.

Statistical analyses were performed using SPSS Statistics (version 25.0, IBM Corp., Armonk, NY, USA). Continuous variables were assessed for normality using visual inspection and the Shapiro-Wilk test. Variables showing approximately normal distributions are reported as mean ± standard deviation, while categorical variables are presented as absolute numbers and percentages. To evaluate factors associated with the predefined primary endpoint of one-year mortality, multivariable logistic regression analysis was performed. The initial multivariable model was intended both to evaluate the association between time-to-surgery and one-year mortality and to identify perioperative factors associated with mortality within this cohort. The regression model included age, ASA classification, treatment modality, grouped time to surgery (< 6 h, 6–12 h, > 12–24 h), antithrombotic therapy, surgical complications (including postoperative hematoma and surgical site infection), and non-surgical complications occurring during hospital stay (urinary tract infection, pneumonia, thrombosis, renal failure, and cardiac events) as independent variables. Results are reported as odds ratios with corresponding 95% confidence intervals. To assess the robustness of the association between time to surgery and one-year mortality, an additional multivariable logistic regression analysis excluding peri- and postoperative complications was performed. This sensitivity model included age, ASA classification, treatment modality, grouped time to surgery, and antithrombotic therapy. In addition, a multivariable Cox proportional hazards regression analysis was performed to account for the timing of death during the one-year follow-up, using the same covariates as the sensitivity model. Patients alive at one year were censored at 365 days. The proportional hazards assumption was tested and was not violated. Cox regression results are reported as hazard ratios with corresponding 95% confidence intervals. A p-value < 0.05 was considered statistically significant.

## Results

### Patient characteristics

The study cohort comprised 1,051 patients with hip fractures (Table 1). The mean age was 80.3 ± 12.5 years, with 66% (*n* = 694) females and 34% (*n* = 357) males. With regard to fracture type, trochanteric fractures were more frequent, accounting for 64.1% (*n* = 674) of cases, whereas femoral neck fractures were observed in 35.9% (*n* = 377). Most patients presented with a substantial preoperative risk profile: ASA score 3 was documented in 66.7% (*n* = 701) and ASA score 4 in 17.6% (*n* = 185). Lower ASA scores were less common, with ASA 1 in 2.5% (*n* = 26) and ASA 2 in 13.2% (*n* = 139) of patients. Surgical management predominantly consisted of intramedullary nailing (60.3%, *n* = 634), followed by arthroplasty (25.3%, *n* = 266) and DHS/FNS fixation (14.4%, *n* = 151).

**Table 1:** General patient data

Variable	n / Mean	SD / %
n	1051	100%
Age [years]	80.3	± 12.5
**Gender**		
male	357	34.0%
female	694	66.0%
**Fracture type**		
Femoral neck	377	35.9%
Trochanteric	674	64.1%
**ASA classification**		
1	26	2.5%
2	139	13.2%
3	701	66.7%
4	185	17.6%
**Treatment**		
Arthroplasty	266	25.3%
DHS/FNS	151	14.4%
Intramedullary nailing	634	60.3%
**Antithrombotic therapy**		
None	515	49.0%
Acetylsalicylic acid	250	23.8%
Platelet Aggregation Inhibitor	64	6.1%
DOAC	168	16.0%
Vitamin K antagonist	54	5.1%
**Time to surgery [h]**	10.2	± 6.2
<6 h	343	30.9%
6-12 h	368	26.4%
>12-24 h	340	31.5%
**Complications**		
Surgical complication	35	3.3%
Hematoma	35	3.3%
Surgical site infection	12	1.1%
**Non-surgical complications**		
Urinary tract infection	170	16.2%
Pneumonia	70	6.7%
Thrombosis	7	0.7%
Renal failure	60	5.7%
Cardiac event	80	7.6%
**Mortality**		
Hospital death	67	6.4%
One-year mortality	310	29.5%
Two-year mortality	421	40.1%

ASA: American Society of Anesthesiologists; DHS: Dynamic Hip Screw; FNS: Femoral Neck System; DOAC: Direct Oral Anticoagulant

### Time to surgery and treatment modalities

The overall mean time to surgery was 10.2 ± 6.2 h. Time to surgery differed significantly between treatment modalities (*p* < 0.001, Kruskal-Wallis test). Patients treated with arthroplasty had a longer time to surgery compared to those treated with DHS/FNS fixation and intramedullary nailing (14.0 ± 6.0 h vs. 9.0 ± 5.0 h vs. 8.0 ± 5.0 h, respectively).

**Fig. 2:**
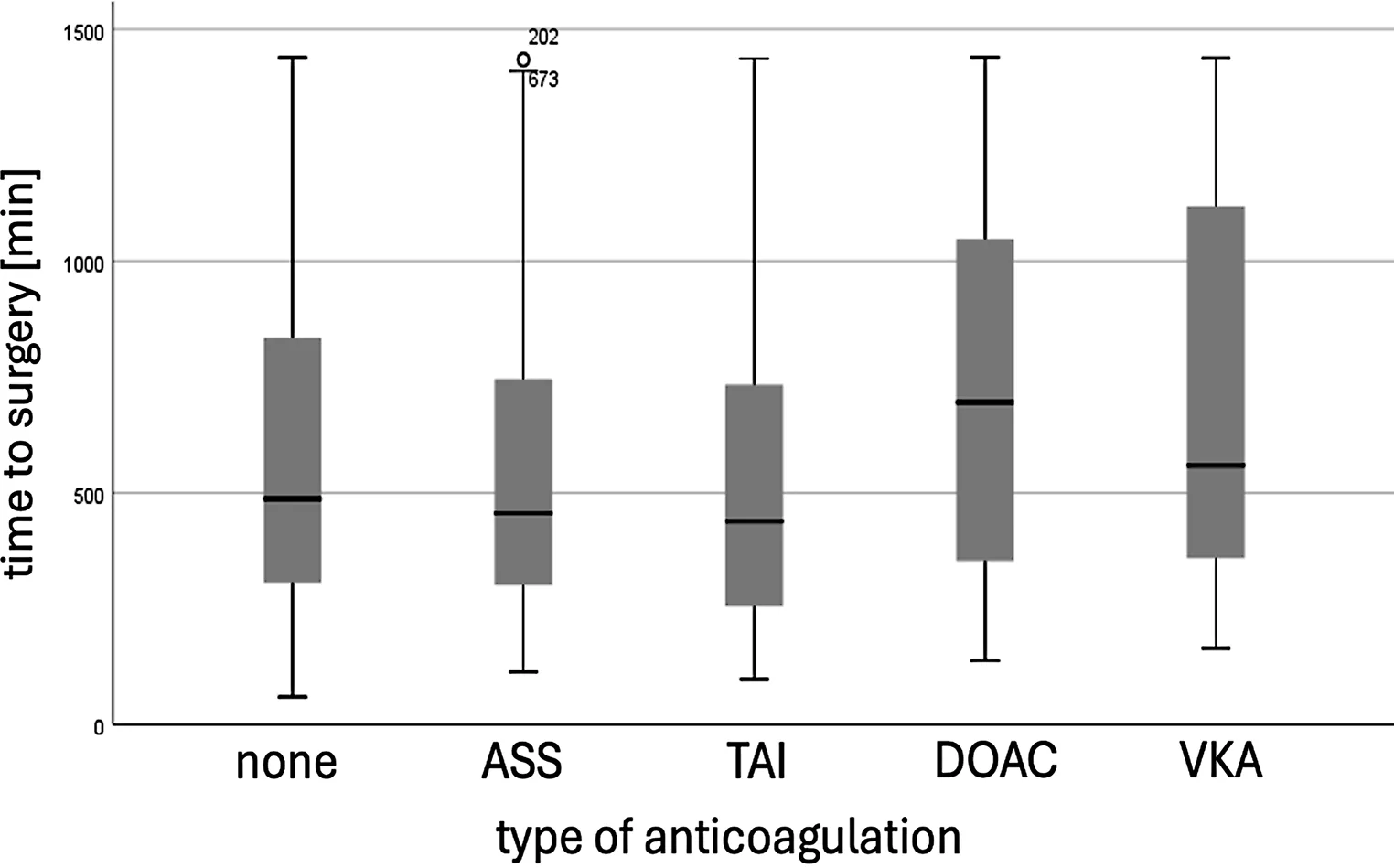
Time to surgery stratified by type of antithrombotic therapy. Box plots illustrating time to surgery stratified by pre-existing antithrombotic therapy: none (*n* = 515), acetylsalicylic acid (ASS; *n* = 250), platelet aggregation inhibitors (TAI; *n* = 64), direct oral anticoagulants (DOAC; *n* = 168), and vitamin K antagonists (VKA; *n* = 54). The central line represents the median, the boxes indicate the interquartile range, and the whiskers denote the range excluding outliers. Time to surgery is displayed in minutes; for reference, 500, 1000, and 1500 min correspond approximately to 8.3, 16.7, and 25 h, respectively

More than half of the cohort was on long-term therapy with antithrombotic agents at the time of admission (51.0%, *n* = 536). Among these patients, acetylsalicylic acid was most frequently used (23.8%, *n* = 250), followed by direct oral anticoagulants (16.0%, *n* = 168), platelet aggregation inhibitors (6.1%, *n* = 64), and vitamin K antagonists (5.1%, *n* = 54). Antithrombotic therapy was not associated with a clinically relevant delay to surgery, with a mean time to surgery of 11.0 ± 6.0 h in patients receiving antithrombotic medication compared to 10.0 ± 6.0 h in patients without antithrombotic therapy (Fig. 2). Significant differences in time to surgery were observed across the different antithrombotic therapy groups (*p* < 0.05, Kruskal-Wallis test,). Post hoc Dunn-Bonferroni comparisons demonstrated that patients receiving direct oral anticoagulants experienced significantly longer times to surgery than the remaining groups. When antithrombotic treatment was analysed as a binary variable (anticoagulation yes vs. no), no significant differences in time to surgery were observed.

### Mortality outcomes

**Fig. 3:**
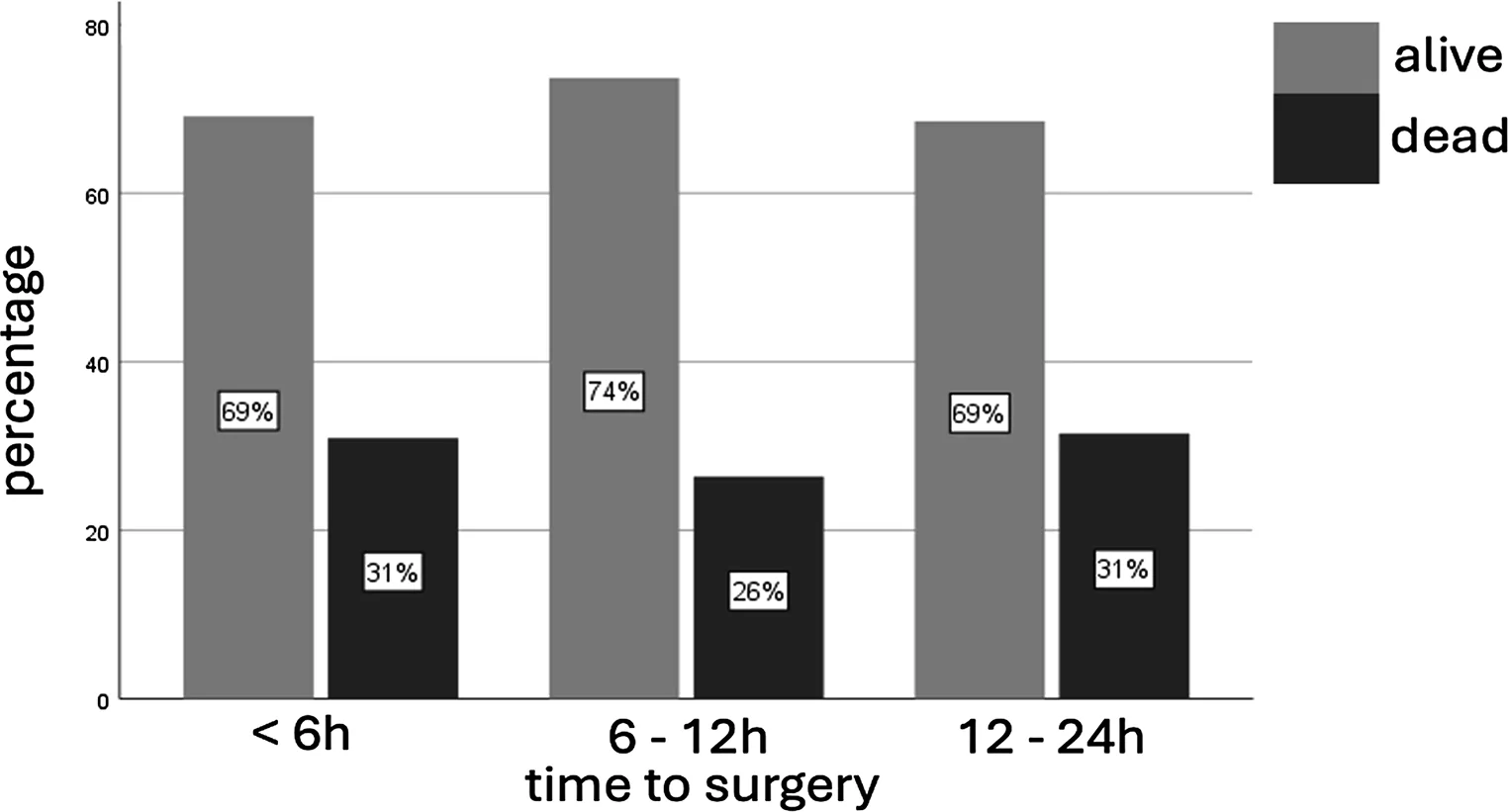
One-year mortality stratified by time-to-surgery intervals. Proportion of patients alive and deceased at one-year follow-up according to time-to-surgery intervals. Mortality was lowest in patients treated within 6–12 h after admission (26.4%, 97/368), compared with < 6 h (30.9%, 106/343) and > 12–24 h (31.5%, 107/340). No statistically significant differences in one-year mortality were observed between the three time intervals (*p* = 0.258)

Overall, in-hospital mortality was 6.4% (*n* = 67). One-year mortality was 29.5% (*n* = 310, Fig. 3) and increased to 40.1% (*n* = 421) at two years. When stratified by time to surgery, patients treated within 6–12 h after admission exhibited the lowest mortality at both follow-up intervals. One-year mortality was 26.4% (97/368) in the 6–12 h group compared with 30.9% (106/343) in patients treated within < 6 h and 31.5% (107/340) in those treated within > 12–24 h. A similar pattern was observed at two years, with the lowest mortality in the 6–12 h group (36.4%, 134/368), compared with the < 6 h group (40.2%, 138/343) and the > 12–24 h group (43.8%, 149/340). Differences in mortality between the three time-to-surgery groups were not statistically significant at one year (*p* = 0.258) or two years (*p* = 0.132, χ² test). No statistically significant differences between the three time-to-surgery groups were observed regarding age (Kruskal-Wallis test), ASA classification, fracture type, treatment modality, or antithrombotic therapy (all *p* > 0.05, χ² tests).

### Impact of surgical and non-surgical complications on mortality

Surgical complications, defined as implant- or procedure-related adverse events, occurred in 3.3% (*n* = 35) of patients and included secondary displacement, dislocation, and implant failure. Postoperative hematoma requiring surgical revision was observed in 3.3% (*n* = 35), while surgical site infection occurred in 1.1% (*n* = 12). In contrast, non-surgical complications were considerably more common. Urinary tract infection was documented in 16.2% (*n* = 170) of patients, pneumonia in 6.7% (*n* = 70), renal failure in 5.7% (*n* = 60), cardiac events in 7.6% (*n* = 80), and thrombosis in 0.7% (*n* = 7).

The multivariable logistic regression model evaluating factors associated with one-year survival was statistically significant (*p* < 0.001, Table 2). Increasing age was independently associated with one-year mortality (OR 1.045, 95% CI [1.018–1.073], *p* = 0.001). Surgical complications, postoperative hematoma, and surgical site infection were not significantly associated with one-year mortality. Likewise, urinary tract infection and thrombosis showed no significant association with survival.

**Fig. 4:**
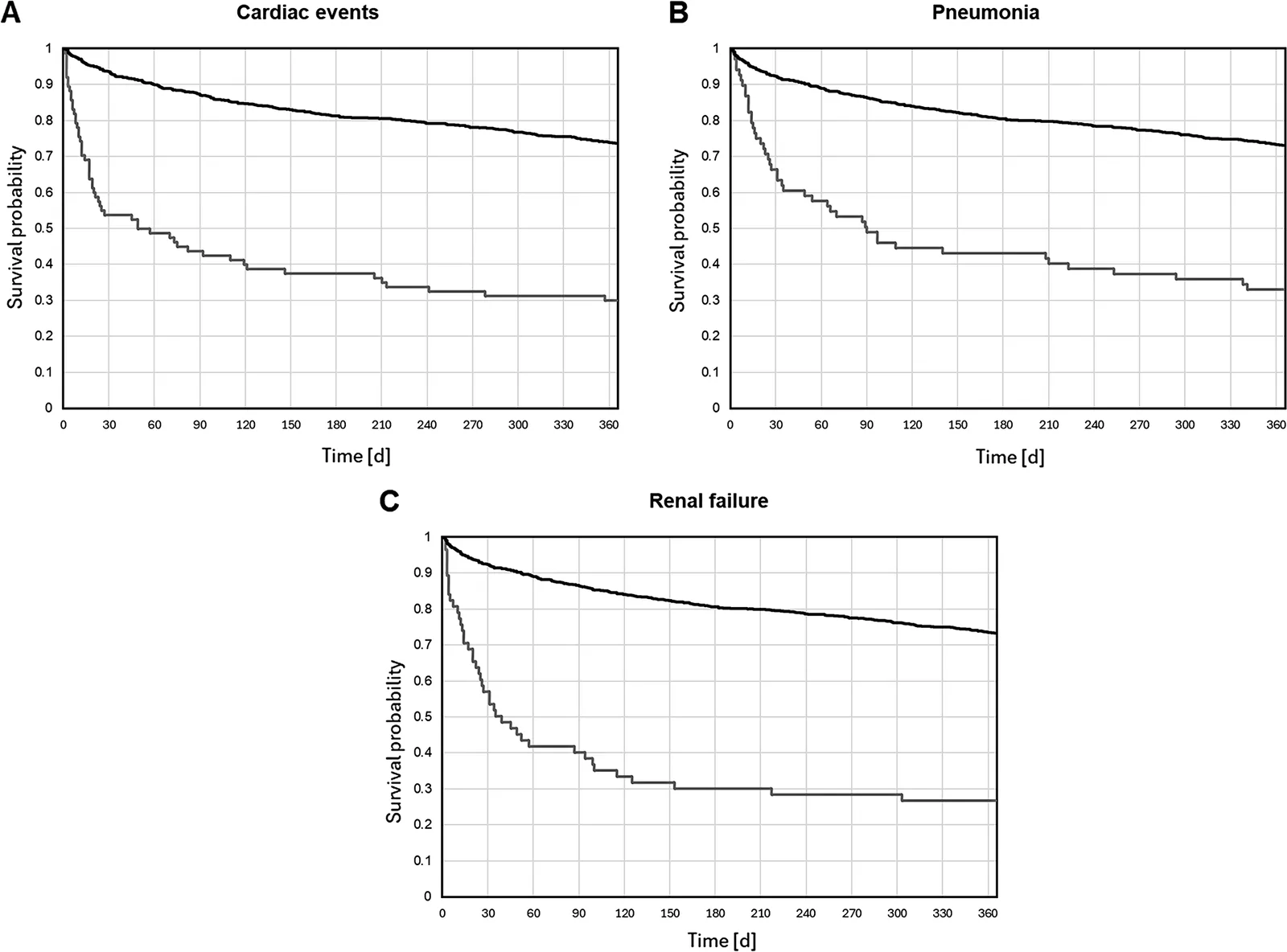
Kaplan-Meier curves for one-year survival according to perioperative non-surgical complications. Kaplan-Meier curves illustrating one-year survival stratified by perioperative non-surgical complications: (**A**) cardiac events, (**B**) pneumonia, and (**C**) renal failure. These complications were significantly associated with one-year mortality in multivariable analysis. In each panel, the black line represents patients without the respective complication, whereas the grey line represents patients with the complication

In contrast, several non-surgical complications emerged as significant independent predictors of increased one-year mortality. Pneumonia showed a strong association with one-year mortality (OR 2.104, 95% CI [1.186–3.734], *p* = 0.011). Renal failure (OR 5.202, 95% CI [1.948–13.891], *p* = 0.001) and cardiac events (OR 5.113, 95% CI [1.935–13.519], *p* = 0.001) demonstrated the strongest associations with one-year mortality (Fig. 4). Neither implant choice nor grouped time to surgery showed a statistically significant association with survival in the adjusted model. A non-significant trend toward improved survival was observed for the 6–12 h time-to-surgery interval compared with the reference category (*p* = 0.078).

In the sensitivity analysis excluding peri- and postoperative complications, time to surgery remained not significantly associated with one-year mortality (overall *p* = 0.133, Supplementary Table S1). Similarly, in the Cox proportional hazards model accounting for the timing of death during one-year follow-up, time to surgery was not significantly associated with mortality (overall *p* = 0.119, Table 3).

**Table 2:** Multivariable logistic regression analysis for one-year mortality

Variable	B	OR	95% CI	*p*-value
**Age**	**0.044**	**1.045**	**[1.018, 1.073]**	**0.001**
*ASA 1* ^*R*^				
ASA 2	-0.901	0.406	[0.083, 1.980]	0.265
ASA 3	0.075	1.078	[0.240, 4.837]	0.922
ASA 4	1.277	3.585	[0.773, 16.644]	0.103
*Endoprothesis* ^*R*^				
DHS/FNS	0.098	1.103	[0.638, 1.908]	0.726
Intramedullary nailing	-0.216	0.806	[0.552, 1.176]	0.263
Surgical complication	-0.627	0.534	[0.189, 1.514]	0.238
Hematoma	0.332	1.394	[0.569, 3.417]	0.468
Surgical site infection	-1.647	0.193	[0.022, 1.679]	0.136
Urinary tract infection	-0.047	0.954	[0.636, 1.430]	0.820
**Pneumonia**	**0.744**	**2.104**	**[1.186**,** 3.734]**	**0.011**
Thrombosis	-0.914	0.401	[0.048, 3.324]	0.397
**Renal failure**	**1.649**	**5.202**	**[1.948**,** 13.891]**	**0.001**
**Cardiac event**	**1.632**	**5.113**	**[1.935**,** 13.519]**	**0.001**
Antithrombotic therapy	0.060	1.062	[0.769, 1.467]	0.716
*Time to surgery > 12–24 h* ^*R*^				
Time to surgery < 6 h	0.242	1.247	[0.861, 1.885]	0.226
Time to surgery 6–12 h	-0.351	0.704	[0.476, 1.040]	0.078

^R^ Reference category. Variables shown in italics represent the reference categories used for the multivariable logistic regression analysis

Boldfaced values indicate significance at *p* < 0.05. B: Regression coefficient; OR: Odds ratio; 95% CI: 95% Confidence interval

ASA: American Society of Anesthesiologists; DHS: Dynamic Hip Screw; FNS: Femoral Neck System

**Table 3:** Multivariable Cox proportional hazards regression analysis for one-year mortality

Variable	B	HR	95% CI	*p*-value
**Age**	**0.036**	**1.037**	**[1.024, 1.051]**	**0.001**
*ASA 1* ^*R*^				
ASA 2	-1.037	0.355	[0.098, 1.287]	0.115
ASA 3	-0.036	0.964	[0.294, 3.162]	0.952
ASA 4	0.922	2.515	[0.752, 8.416]	0.134
*Endoprothesis* ^*R*^				
DHS/FNS	0.067	1.069	[0.706, 1.619]	0.753
Intramedullary nailing	-0.048	0.954	[0.724, 1.256]	0.735
Antithrombotic therapy	-0.066	0.936	[0.738, 1.188]	0.588
*Time to surgery > 12–24 h* ^*R*^				
Time to surgery < 6 h	-0.213	0.808	[0.607, 1.077]	0.146
Time to surgery 6–12 h	0.069	1.071	[0.805, 1.425]	0.638

^R^ Reference category. Variables shown in italics represent the reference categories used for the multivariable Cox proportional hazards regression analysis

Boldfaced values indicate significance at *p* < 0.05. B: Regression coefficient; HR: Hazard ratio; 95% CI: 95% Confidence interval

ASA: American Society of Anesthesiologists; DHS: Dynamic Hip Screw; FNS: Femoral Neck System

## Discussion

### Principal findings

Within this consecutive single-centre cohort of 1,051 patients with hip fractures managed operatively within 24 h of admission, we observed no statistically significant differences in one- or two-year mortality across the examined time-to-surgery intervals (< 6 h, 6–12 h, > 12–24 h). Although the lowest mortality was observed in the 6–12 h interval (26.4% vs. 30.9% and 31.5%, respectively), these differences did not reach statistical significance. Overall, one-year mortality remained considerable at 29.5% despite uniformly early surgical treatment within 24 h. This finding remained consistent in the sensitivity analysis excluding peri- and postoperative complications and in the time-to-event analysis using Cox proportional hazards regression.

In contrast, medical (non-surgical) complications showed the strongest independent associations with one-year mortality, with particularly strong associations observed for pneumonia (OR = 2.1), renal failure (OR = 5.2), and cardiac events (OR = 5.1). These findings suggest that, within an established early-treatment framework, outcomes are less dependent on marginal differences in surgical timing. Instead, outcomes appear more strongly linked to the prevention, early recognition, and management of perioperative medical complications. In this context, an orthogeriatric co-management may contribute to improved outcomes, as suggested by previous studies [12]. In a recent systematic review, Mazarello et al., demonstrated that early comprehensive geriatric assessment is consistently associated with improved functional outcomes, reduced complication rates, shorter hospital stays, and lower mortality in hip fracture patients [13].

### Timing of surgery and mortality – Evidence base

The relationship between time to surgery and mortality after hip fracture has been extensively investigated, yet remains heterogeneous [6]. Large database analyses have demonstrated that prolonged surgical delay, particularly beyond 48 h after admission, is associated with increased mortality, complications, and healthcare resource utilisation [14]. A comprehensive meta-analysis by Chang et al., including more than 25,000 patients, identified surgical delay beyond 48 h as a significant risk factor for mortality (OR 1.91), whereas no statistically significant association was observed for delays beyond 24 h [15]. In contrast, another meta-analysis of Welford et al. reported lower mortality with surgery within 24 h compared with later time points [8], and large population-based data of Pincus et al. suggest clinically relevant threshold effects around 24 h for short-term mortality and complications [16].

Our results are consistent with this interpretation. Within a strictly implemented 24-hour treatment policy, no significant differences in one-year mortality were observed across different time-to-surgery intervals. This suggests that once surgery is performed within an established early timeframe, further reduction to an ultra-early approach may not confer additional survival benefit. Supporting this concept, Ryan et al., in an analysis of more than two million patients from the US National Inpatient Sample (NIS), demonstrated that next-day surgery was not associated with increased in-hospital mortality, whereas procedures performed two or more calendar days after admission were linked to significantly higher mortality, effectively defining clinically relevant delay as ≥ 2 days [17].

Randomized evidence points in a similar direction. In the HIP ATTACK trial, accelerated surgery targeting treatment within 6 h after diagnosis did not significantly reduce mortality compared with standard care, although benefits were observed for selected secondary outcomes, including delirium and urinary tract infections [18]. Together, these findings suggest that clinically meaningful increases in mortality primarily occur beyond established early-treatment thresholds, whereas further acceleration within an already implemented 24-hour treatment pathway may not confer additional survival benefit.

### Confounding by indication and interpretation of timing effects

Interpretation of timing effects is inherently complicated by confounding by indication. Patients requiring preoperative stabilization – such as management of anticoagulation, cardiopulmonary optimisation, or treatment of acute medical conditions – are more likely to experience surgical delay and simultaneously carry a higher baseline mortality risk.

In the present cohort, variability in time-to-surgery was limited to a relatively narrow range within 24 h, reducing the potential for differential treatment allocation. While residual confounding cannot be excluded, the absence of a timing effect within this controlled framework supports the interpretation that small differences in surgical timing are unlikely to be a dominant determinant of survival. Recent analyses have further highlighted the importance of patient-related factors such as frailty and cardiovascular risk profiles when interpreting the relationship between surgical delay and outcomes after hip fracture [19]. This supports the notion that observed timing effects may partly reflect underlying patient vulnerability rather than the effect of surgical timing alone.

### Role of perioperative complications and orthogeriatric factors

In contrast to timing itself, several non-surgical complications were strongly associated with increased one-year mortality. Pneumonia, renal failure, and cardiac events were independently associated with one-year mortality, each demonstrating substantial effect sizes. These findings may underline the central importance of perioperative orthogeriatric co-management in this vulnerable population. While surgical complications requiring revision were infrequent and not independently associated with mortality, systemic complications had a pronounced impact on long-term survival. This highlights that hip fracture outcomes are not determined solely by operative timing or implant choice but are strongly influenced by frailty, comorbidity burden, and postoperative medical events.

Chang et al. demonstrated that pulmonary disease (OR 1.52) and cardiovascular disease (OR 1.14) were independently associated with increased mortality; however, these variables predominantly reflect pre-existing comorbidities rather than acute postoperative events [15]. In contrast, our analysis focused on complications that developed during the perioperative course, with pneumonia and cardiac events emerging as strong independent predictors of one-year mortality. Although these findings are not directly comparable due to the different definitions of risk factors, they consistently underline the major impact of cardiopulmonary vulnerability on survival. Notably, Simunovic et al. reported that prolonged time to surgery was associated with an increased risk of postoperative pneumonia, suggesting that surgical delay may contribute to mortality indirectly by increasing the likelihood of acute medical complications [6]. Our findings therefore support the concept that optimisation of perioperative medical care may be as important as adherence to strict timing targets.

Notably, inspection of the Kaplan-Meier curves (Fig. 4) indicates a pronounced early separation of the survival curves in patients who developed pneumonia, renal failure, or cardiac events. A substantial proportion of deaths occurred within the first 30–60 days after the complication, suggesting that these events are closely linked to early excess mortality. This temporal pattern further supports the potential relevance of early, structured orthogeriatric management and proactive prevention of medical complications during the acute phase of care.

### Clinical implications under mandatory 24-h treatment policies

In Germany, mandatory national regulations now require operative treatment of hip fractures within 24 h of admission [11]. Such policies aim to standardise care and reduce delays but also impose logistical and clinical pressures on treating teams.

The present data suggest that compliance with a 24-hour treatment target is appropriate and consistent with international recommendations. However, the results do not support the assumption that ultra-early surgery within a few hours after admission necessarily translates into improved long-term survival within such a system. Clinical decision-making should therefore prioritise timely but medically optimised surgery rather than acceleration at the expense of adequate preparation. Particularly in patients with complex comorbidities careful preoperative management may be more relevant for survival than minimal reductions in time-to-surgery within the 24-hour window. Notably, more than half of the patients in the present cohort were receiving antithrombotic medication at admission and were not routinely excluded from early surgical treatment. Although patients receiving direct oral anticoagulants experienced slightly longer times to surgery, antithrombotic therapy was not independently associated with one-year mortality. This finding may be of interest in the ongoing discussion regarding the perioperative management of anticoagulated patients with hip fractures.

### Limitations

Several limitations must be acknowledged. First, this study has a retrospective design and is therefore subject to inherent limitations, including potential information bias and unmeasured confounding. Second, the analysis was conducted at a single level-one trauma centre with an established early-treatment pathway. Although this ensures internal consistency of care, generalisability to other healthcare systems may be limited. Third, confounding by indication cannot be excluded. It remains possible that patients with greater medical stability were prioritised for earlier surgery, whereas those requiring preoperative optimization – such as reversal of anticoagulation or cardiopulmonary stabilization – experienced slight delays within the 24-hour window.

Although multivariable regression was performed to adjust for measured confounders, residual confounding due to unmeasured variables cannot be excluded. In addition, ASA classification was used as the primary measure of preoperative risk but does not fully capture comorbidity burden, frailty, pre-fracture mobility, cognitive status, residential status, or other geriatric risk factors. These variables were not routinely collected in the original retrospective dataset and were therefore unavailable for inclusion in the multivariable analyses.

Perioperative complications were included in the initial multivariable model to explore factors associated with one-year mortality. As these events may occur after treatment exposure and may lie on the causal pathway between surgical timing and mortality, their inclusion may introduce overadjustment or mediation bias. However, the additional sensitivity analysis excluding peri- and postoperative complications yielded consistent findings regarding the absence of a significant association between time to surgery and one-year mortality. Nevertheless, given the observational study design and potential residual confounding, the regression results should be interpreted as associations rather than causal effects.

Finally, while complete follow-up of vital status was ensured through registry linkage, the study focused primarily on mortality outcomes. Functional recovery and quality-of-life measures were not assessed. This is an important limitation, as evidence from a systematic review by Dyer et al. demonstrates that hip fracture has a substantial and sustained impact on patient-centred outcomes. Only 40–60% of patients regain their pre-fracture level of mobility, and a considerable proportion remain dependent in activities of daily living even one to two years after injury [20]. Accordingly, mortality alone does not adequately reflect the full burden of disease or the effectiveness of treatment strategies in this population.

## Conclusion

Within an implemented 24-hour treatment pathway, further acceleration towards ultra-early surgery was not associated with improved survival in this cohort of patients with hip fractures. Instead, perioperative medical complications were strongly associated with mortality. These findings do not challenge the importance of timely surgery but suggest that, within an established 24-hour pathway, comprehensive perioperative and orthogeriatric management may be at least as important as further reductions in time to surgery.

## Supplementary Information

Below is the link to the electronic supplementary material.

Supplementary Material 1

## Data Availability

The datasets generated and/or analysed during the current study are not publicly available due to institutional data protection regulations but are available from the corresponding author on reasonable request.
