# Supplementary material for: Hip fracture surgery within 24 hours: no association between time to surgery and mortality in a real-world cohort
Source: Eur J Trauma Emerg Surg. 2026 Sep 15;52(1):290. doi: 10.1007/s00068-026-03331-0 (PMC13577985; doi:10.1007/s00068-026-03331-0)
Supplement: Supplementary file 1 [file 68_2026_3331_MOESM1_ESM.docx]

Supplementary Table 1. Sensitivity analysis of factors associated with one-year mortality using multivariable logistic regression excluding peri- and postoperative complications

| **Variable** | **B** | **OR** | **95% CI** | **p-value** |  |
| --- | --- | --- | --- | --- | --- |
| **Age** | **0.047** | **1.048** | **[1.032, 1.065]** | **0.001** |  |
| ASA |  |  |  | **0.001** |  |
| *ASA 1^R^* | *-* | *1.000* | *Reference* | *-* |  |
| ASA 2 | -1.150 | 0.317 | [0.077, 1.305] | 0.111 |  |
| ASA 3 | -0.072 | 0.930 | [0.249, 3.437] | 0.915 |  |
| ASA 4 | 1.185 | 3.269 | [0.851, 12.573] | 0.085 |  |
| Treatment modality |  |  |  | 0.519 |  |
| *Endoprothesis^R^* | *-* | *1.000* | *Reference* | *-* |  |
| DHS/FNS | 0.132 | 1.141 | [0.676, 1.926] | 0.623 |  |
| Intramedullary nailing | -0.121 | 0.886 | [0.623, 1.261] | 0.500 |  |
| Antithrombotic therapy |  |  |  |  |  |
| *Yes^R^* | *-* | *1.000* | *Reference* | *-* |  |
| No | -0.111 | 0.895 | [0.660, 1.213] | 0.473 |  |
| Time to surgery |  |  |  | 0.133 |  |
| *Time to surgery >12-24 h^R^* | *-* | *1.000* | *Reference* | *-* |  |
| Time to surgery <6 h | -0.208 | 0.812 | [0.565, 1.167] | 0.261 |  |
| Time to surgery 6-12 h | 0.154 | 1.166 | [0.805, 1.690] | 0.416 |  |
| ^R^ Reference category. Variables shown in italics represent the reference categories used for the multivariable logistic regression analysis. For categorical variables, p-values shown in the main variable row represent the overall Wald test. Boldfaced values indicate significance at p < 0.05.  B: Regression coefficient; OR: Odds ratio; 95% CI: 95% Confidence interval.  ASA: American Society of Anesthesiologists; DHS: Dynamic Hip Screw; FNS: Femoral Neck System. | | | | | |
